# Klotho antiaging protein: molecular mechanisms and therapeutic potential in diseases

**DOI:** 10.1186/s43556-025-00253-y

**Published:** 2025-03-22

**Authors:** Aditya Dipakrao Hajare, Neha Dagar, Anil Bhanudas Gaikwad

**Affiliations:** Department of Pharmacy, Birla Institute of Technology and Science Pilani, Pilani Campus, Rajasthan, 333031 India

**Keywords:** Klotho, Diabetic kidney disease, Anti-aging, Cardiovascular diseases, Alzheimer's disease

## Abstract

Klotho, initially introduced as an anti-aging protein, is expressed in the brain, pancreas, and most prominently in the kidney. The two forms of Klotho (membrane-bound and soluble form) have diverse pharmacological functions such as anti-inflammatory, anti-oxidative, anti-fibrotic, tumour-suppressive etc. The membrane-bound form plays a pivotal role in maintaining kidney homeostasis by regulating fibroblast growth factor 23 (FGF 23) signalling, vitamin D metabolism and phosphate balance. Klotho deficiency has been linked with significantly reduced protection against various kidney pathological phenotypes, including diabetic kidney disease (DKD), which is a major cause of chronic kidney disease leading to end-stage kidney disease. Owing to the pleiotropic actions of klotho, it has shown beneficial effects in DKD by tackling the complex pathophysiology and reducing kidney inflammation, oxidative stress, as well as fibrosis. Moreover, the protective effect of klotho extends beyond DKD in other pathological conditions, including cardiovascular diseases, alzheimer's disease, cancer, inflammatory bowel disease, and liver disease. Therefore, this review summarizes the relationship between Klotho expression and various diseases with a special emphasis on DKD, the distinct mechanisms and the potential of exogenous Klotho supplementation as a therapeutic strategy. Future research into exogenous Klotho could unravel novel treatment avenues for DKD and other diseases.

## Introduction

In 1997, the discovery of Klotho led to its identification as a potential anti-aging protein with broad therapeutic implications [1]. Klotho exists in three subfamilies, including α-Klotho, β-Klotho, and γ-Klotho, where α-Klotho has two distinct forms (membrane-bound and soluble form). The membrane-bound form serves a role in kidney fibroblast growth factor 23 (FGF 23) signalling, which in turn regulates phosphate homeostasis and vitamin D metabolism [2]. The soluble form acts as a circulating hormone exhibiting diverse activities, such as anti-inflammatory [3, 4], anti-oxidative stress [5], tumour-suppressive [6, 7], and proteolytic cleavage activity [8].

The reduction in klotho levels has been implicated in a variety of kidney pathological conditions, including diabetic kidney disease (DKD), which is the most prevalent cause of end-stage kidney failure, affecting 30–40% of individuals globally with type 1 or type 2 diabetes mellitus (DM) [9, 10]. A report has shown that excessive production of Klotho rescued mice against kidney disease and longer their existence by approximately 30% [11]. Klotho safeguards the pancreatic islets of Langerhans activity, also boosting insulin production and decreasing blood glucose levels, which directly impacts slower kidney injury progression [12]. It also shows a beneficial role in the calcium and phosphate metabolism phenomenon. Exogenous Klotho protein supplementation inhibits oxidative stress production and hyperglycemia, correlating with different kidney injury studies. However, a thorough investigation of the impact of exogenous Klotho on DKD is still needed. As DKD progresses, multitudinous metabolic and hemodynamic overloads develop, including hyperfiltration, ischemia, and hypoxia, followed by an increase in the production of reactive oxygen species (ROS) [13, 14]. Furthermore, these overloads can lead to malfunction and injury to cells, trigger an inflammatory response, and induce subsequent fibrosis, ultimately causing kidney injury. Numerous medication classes of drugs such as hypoglycemic agents, angiotensin-converting enzyme (ACE) inhibitors and angiotensin II receptor blockers (ARBs), renin-angiotensin system (RAS) blockers, sodium-glucose cotransport protein-2 inhibitors (SGLT2i), and glucagon-like peptide-1 receptor (GLP1R) agonists are available in the market to beneficially cure diabetes and DKD [15]. However, the risk of DKD might increase in individuals suffering from fleetingly hyperglycemia condition, also in cases where medication keeps the glycemic activity largely within the usual level [16, 17]. Supplementing with exogenous Klotho protein retained endogenous kidney expression of Klotho while raising serum Klotho levels, demonstrating protective benefits on the kidney’s anatomy and function. Still, the pathophysiology of DKD is not well known, and existing medicines cannot entirely address the condition.

The various cross-sectional studies confirm that inadequate glycemic management is the primary risk factor for DKD, which is also a growing worldwide health concern. The Diabetes Control and Complications Trial (DCCT) in T1D patients and the U.K. Protective Diabetes Study (UKPDS) in T2D patients demonstrated that reducing hyperglycemia resulted in a decreased risk of DKD [18]. In addition to hyperglycemia, other risk factors that contribute to the development of DKD include dyslipidaemia, alcohol, smoking, hereditary disorders, microalbuminuria, obesity, and dietary factors (Table 1). Hence, in this case, exogenous Klotho might be the drug of choice, considering the role of Klotho in improving insulin sensitivity, reducing blood glucose levels, and protecting kidneys from further damage.

**Table 1:** Risk factors for DKD

Risk factors	Regulatory pathways	Effects on kidney	References
Hyperglycemia	Upregulated NF-κB (Nuclear Factor Kappa B)	Increases pro-inflammatory response and decreases Klotho's levels due to hyperglycemia, which might potentially cause kidney damage	[19, 20]
	Increased oxidative stress, apoptosis, mitogen-activated protein kinase (MAPK) expression	Hyperglycemia in cultured podocytes causes dysregulation of Klotho expression, reduced GFR (Glomerular Filtration Rate), elevated ROS levels, and apoptotic markers which in turn causes podocyte lytic death in DKD	[21]
	Increased advanced glycation end-products (AGEs), protein kinase C (PKC) and upregulated renin–angiotensin–aldosterone system (RAAS)	The concentration of Klotho protein is disrupted, vitamin D receptor function is suppressed, and intracellular metabolism and cellular processes are altered by hyperglycemia, which is linked to DKD	[22, 23]
Dyslipidemia	Decreased fat oxidation, downregulation of ACOX1 and CPT1 gene expression	In the obese mice model, disruptions to the hepatic lipid metabolism and lipid buildup lead to a direct relation to suppression of Klotho expression	[24]
	Increased type 1 insulin-like growth factor receptor (IGF-1R)/Ras-related C3 botulinum toxin substrate 1 (RAC1)/LOX-1 (lectin-like oxidized low-density lipoprotein receptor-1 OLR-1) signal	Both in vivo and in vitro models of DKD show podocyte damage due to glomerular ox-LDL accumulation, which downregulates Klotho expression and impairs kidney lipid metabolism eventually culminating in dyslipidemia	[25]
	-	Klotho concentrations were shown to be negatively correlated with triglyceride levels and a probability of hyperlipidemia in Americans	[26]
Obesity	-	The results of cross-sectional research involving 2989 people with type 2 diabetes showed that blood levels of s-Klotho were inversely correlated with obesity and CVD and directly linked with eGFR and HbA1c levels	[27]
	-	Particularly in older, obese, and diabetic individuals, serum-soluble Klotho levels were significantly correlated with eGFR and adversely connected with the occurrence of CKD	[28]
Smoking	-	Based on the statistical study, men with CKD from the United States who quit smoking had considerably lower blood α-Klotho levels than those who never smoked	[29]
	-	Individuals who did not smoke or had not developed CKD were more likely to have greater Klotho concentrations and lower levels of uric acid, serum phosphorus, vitamin D, and blood urea nitrogen (BUN)	[30]
Genetic	Upregulated transforming growth factor β1 (TGF β1), mTOR signalling, kidney hypertrophy	Klotho gene deficiency causes the destruction of kidney function and structure which makes kidneys more vulnerable to diabetic damage and exacerbates early DKD in mice model	[2]
	Increased oxidative stress, lipid peroxidation, mitochondrial damage	Genetic changes in the glomerular and tubulointerstitial compartments are the outcome of ICR-derived glomerulonephritis (ICGN) mice lacking the Klotho gene. These mice also have increased production of 4-hydroxy-2 nonenal-positive cells, apoptotic nuclei, urinary excretion of 8-hydroxydeoxyguanosine, and induction of mtDNA mutations	[31]
	Increased oxidative stress, inflammation	In addition to causing cardiovascular and non-cardiovascular risk, Klotho genetic variants may also have an impact on clinical connections and may be linked to inflammation, salt sensitivity, hypertension, and CKD	[32]
Alcohol	Increased insulin resistance, malfunctioning β-cells in the pancreas	Long-term alcohol use raises the risk of hyperglycemia resulting in type 2 diabetes mellitus (T2DM), which may be linked to a reduction in Klotho expression	[33]
Dietary factors	Increased oxidative stress, pro-inflammatory proteins	Epidemiological reports verified that in chronic inflammatory disorders, higher consumption of dietary fiber counteracts a decrease in Klotho levels	[34, 35]
	-	Increasing dietary fiber consumption in older participants in the US can significantly boost Klotho protein levels in patients suffering from aging-related illnesses such as chronic kidney disease, cardiovascular disease, and obesity-related disease	[36]
	Reduced GFR	In contrast, patients suffering from chronic kidney illness revealed that those with high dietary phosphorus consumption exhibited considerably decreased levels of Klotho, which in turn led to kidney tubular damage	[37]
	Reduced pro-inflammatory proteins	Anti-inflammatory diets appear to raise Klotho levels via regulating chronic inflammation, which includes kidney disease and diabetes, whereas pro-inflammatory diets are associated with lower S-Klotho plasma levels in youngsters	[38–40]

The mechanistic and therapeutic role of Klotho extends beyond DKD. The systemic effects of Klotho indicate its implication in several disease pathophysiologies other than kidney, including cardiovascular diseases, alzheimer’s disease, cancer, inflammatory bowel disease, liver disease and aging-associated diseases. The ability of Klotho to modulate oxidative stress, inflammation, and metabolic dysregulation highlights its broader therapeutic potential, making it a promising candidate for combating DKD and other diseases. Therefore, the present review discusses the crucial role of klotho in various diseases, including cardiovascular, alzheimer’s disease, and aging-associated diseases, with a special emphasis on DKD from the mechanistic and therapeutic viewpoint along with insights into klothos’ molecular structure and localization.

## Molecular structure and localization of Klotho

The Klotho protein derives its name from Klotho (Clotho), a figure in Greek mythology and one of the Moirai, who was thought to weave the thread of life and govern human fate. It was initially reported by Kuro-o, who discovered that a transgenic insertional mutation in mice disrupted the Klotho gene, which encodes Klotho, resulting in an abnormally early aging phenotype [1]. The human α-klotho gene has five exons and fifty thousand base pairs, found on the 13q12 chromosome. It exists in several body parts, with the kidney containing the greatest amount and its product. In comparison, β-Klotho shares many similarities with α-Klotho regarding size and structure and is found on the fourth chromosome. Its primary source is adipose tissue, but skeletal muscle, the aorta, and the heart contain its messenger RNA [41]. Lctl (lactamase-like gene) encodes the third member of the Klotho family. A transmembrane lactase-like protein, also known as γ-klotho or lactase-phlorizin hydrolase-related protein, is the product of this process. Most organs exhibit it at a low level, found on chromosome 15. The maximum and minimum expression levels are shown by α-klotho and γ-klotho, respectively [1, 41–43].

Both humans and mice have been shown to contain two types of Klotho: the secreted and the full-length transmembrane. An intracellular domain, a single-pass membrane domain, and an extracellular domain with two interior repetitions (KL1 and KL2) comprise the full-length transmembrane Klotho. These KL1 and KL2 extracellular domains are essential to Klotho's primary activity and functions [12, 44–46]. The kidney is the primary source of secreted Klotho and the main product of the KL gene in humans [45]. Complete transmembrane Klotho and soluble 130 kDa Klotho protein are crucial co-receptors for FGF23 [46]. Klotho builds a complex with FGFR1c, FGFR3c, and FGFR4 to improve the binding capacity of FGFRs specifically to FGF23 at target organs. Consequently, Klotho transforms canonical FGFRs into a particular FGF23 receptor. Because of this connection, Klotho is obligated to metabolise calcium, phosphate, and vitamin D [12]. The kidney's proximal tubular cells are principally accountable for a vital mineral balance.

FGF23 resistance in Klotho-deficient mice leads to abnormal calcifications of soft tissues and blood vessels in response to elevated serum calcitriol levels, which enhance dietary Ca2 + absorption and hinder phosphate elimination in the urine [47]. However, Klotho is maximum expressed in specific organs such as the kidney tubules, parathyroid glands, and the brain's choroid plexus and is minimum observed in the bone, placenta, prostate, and small intestine [45, 48, 49]. Mutations in the Klotho gene result in dysfunctions in nearly every organ and tissue. Therefore, one of the body's most essential attributes could involve the maintenance of the klotho protein in various diseases.

## Role of Klotho in aging

Aging is not only a compilation of ailments that occur in the later stages of life; it is a dynamic process that unfolds throughout the lifespan. The escalating issue of an aging population is a significant economic, social, and medical concern of modern society. Over time, aging causes a segmental and gradual loss of strength and biological function, which leads to a decline in resistance and increasing physiological weakness [50–52]. Multiple biochemical pathways actively control aging. It is distinguished by a number of molecular and cellular features, including abnormal nutrition sensing, mitochondrial malfunction, cellular senescence, epigenetic imbalance, and loss of connectivity between cells [51, 53]. Globally, chronic illnesses tend to be more common in the aging population. Chronic illnesses need lengthy therapy, which alters the character of healthcare facilities and raises demand for them.

On the other hand, Klotho is an anti-aging protein with diverse therapeutic roles in the pathophysiology of different organs, such as the kidneys and skeletal muscle. Numerous pathways implicated in aging processes are regulated by Klotho, including Wnt signaling, insulin signaling, and phosphate homeostasis. It also impacts intracellular signaling pathways, including TGF-β, p53/p21, cAMP, and protein kinase C (PKC) [51, 54, 55]. Klotho expression and circulation levels decrease with increasing age. Klotho-deficient mice have excessive phosphate levels because of phosphate excretion imbalance in the urine. However, they also exhibit a complicated phenotype that includes stunted development, atrophy of several organs, hypercalcemia, kidney fibrosis, cardiac hypertrophy, and reduced lifespans [1, 55, 56]. Given that supplementation or Klotho gene expression has been shown to suppress and repair Klotho-deficient phenotypes, it is likely that Klotho might have a protective impact against aging illness.

Recent cross-sectional cohort research with 346 healthy individuals aged 18 to 85 years showed that serum Klotho levels are negatively correlated with age and that older individuals (ages 55 to 85) exhibited the lowest serum α-Klotho levels [57]. Another observational cohort research, which had around 804 adults over 65 years old, was conducted in Italy and found a negative correlation between serum Klotho levels and all-cause mortality [58]. Furthermore, those with decreased serum Klotho levels had a comparable increased risk for all-cause death, according to a meta-analysis of six cohort studies that included adult CKD patients [59]. Additionally, preclinical research has demonstrated that overexpressing the Klotho gene in transgenic mice can postpone or reverse aging [60]. Therefore, increasing Klotho levels emerges as a promising strategy in DKD, CKD, and aging disorders.

## Role of Klotho in diabetic kidney disease

**Fig. 1:**
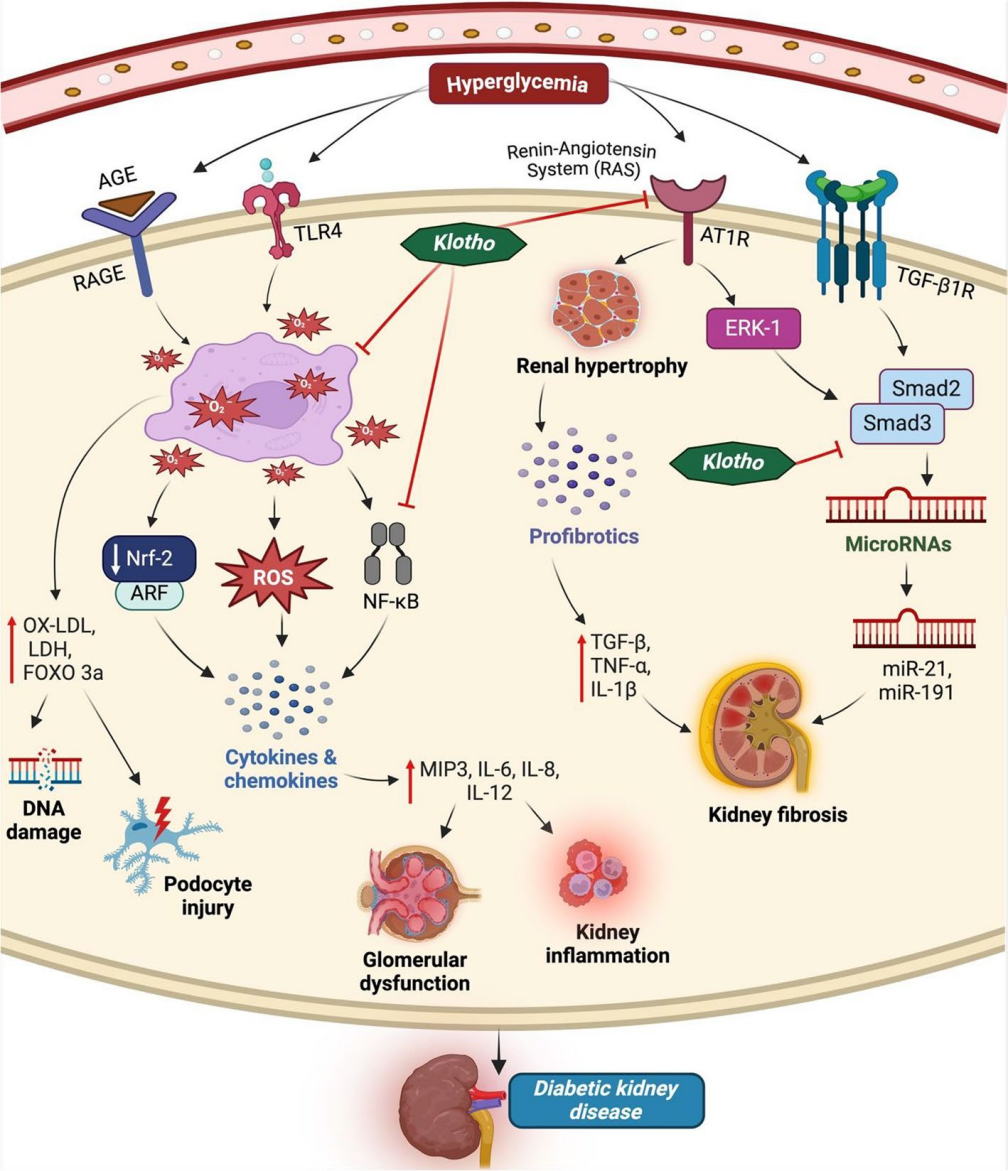
Klotho inhibits oxidative stress, inflammation, and fibrosis pathways implicated in diabetic kidney disease (DKD). Hyperglycemia is a major factor in DKD. In these circumstances, TLR is activated directly, whereas AGE activates RAGE. Both heighten oxidative stress, which inhibits Nrf-2 function and raise NF-κB expression, resulting in the release of stress markers and cytokines, which subsequently cause kidney damage and inflammation. RAS activates AT1R, increasing kidney burden and secreting profibrotic mediators, leading to kidney fibrosis. Micro-RNAs like miR-21 and miR-191 are released when Smad3 is stimulated by TGF-β1R. However, Klotho shows anti-oxidant, anti-inflammatory, and anti-fibrotic actions in DKD Abbreviations: TLR, toll-like receptor; AGE, advanced glycation end products; RAGE, receptor for advanced glycation end products; FOXO3, forkhead Box O3; OX-LDL, oxidized low-density lipoprotein (Ox-LDL); MIP 3, macrophage Inflammatory protein-3; Smad3, suppressor of mother against decapentaplegic

**Fig. 2:**
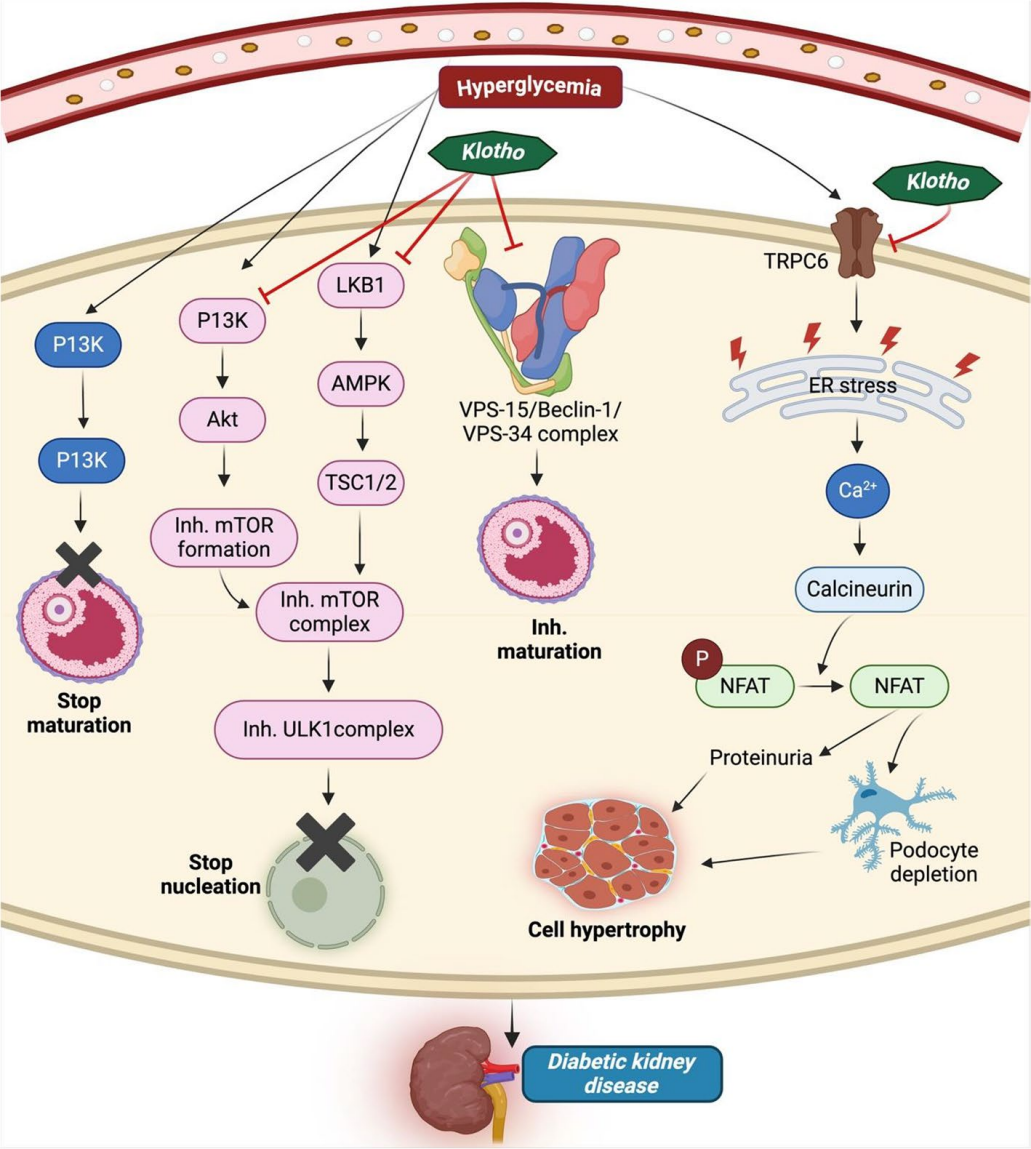
Klotho regulates autophagy and calcium balance in diabetic kidney disease. Cell growth is suppressed by directly activating ATG and Beclin-1 while inhibiting PI3K and LKB expression. TRPC6 induces ER stress, enhancing calcium production and activating calcineurin that dephosphorylates the NFAT, leading to cell hypertrophy. However, Klotho maintains cell growth and calcium balance in DKD Abbreviations: ATG, autophagy-related genes; PI3K, phosphoinositide 3-kinase; AMPK, targeting AMP-activated protein kinase; TRPC6, transient receptor potential canonical 6; NFAT, nuclear factor of activated T cells

**Fig. 3:**
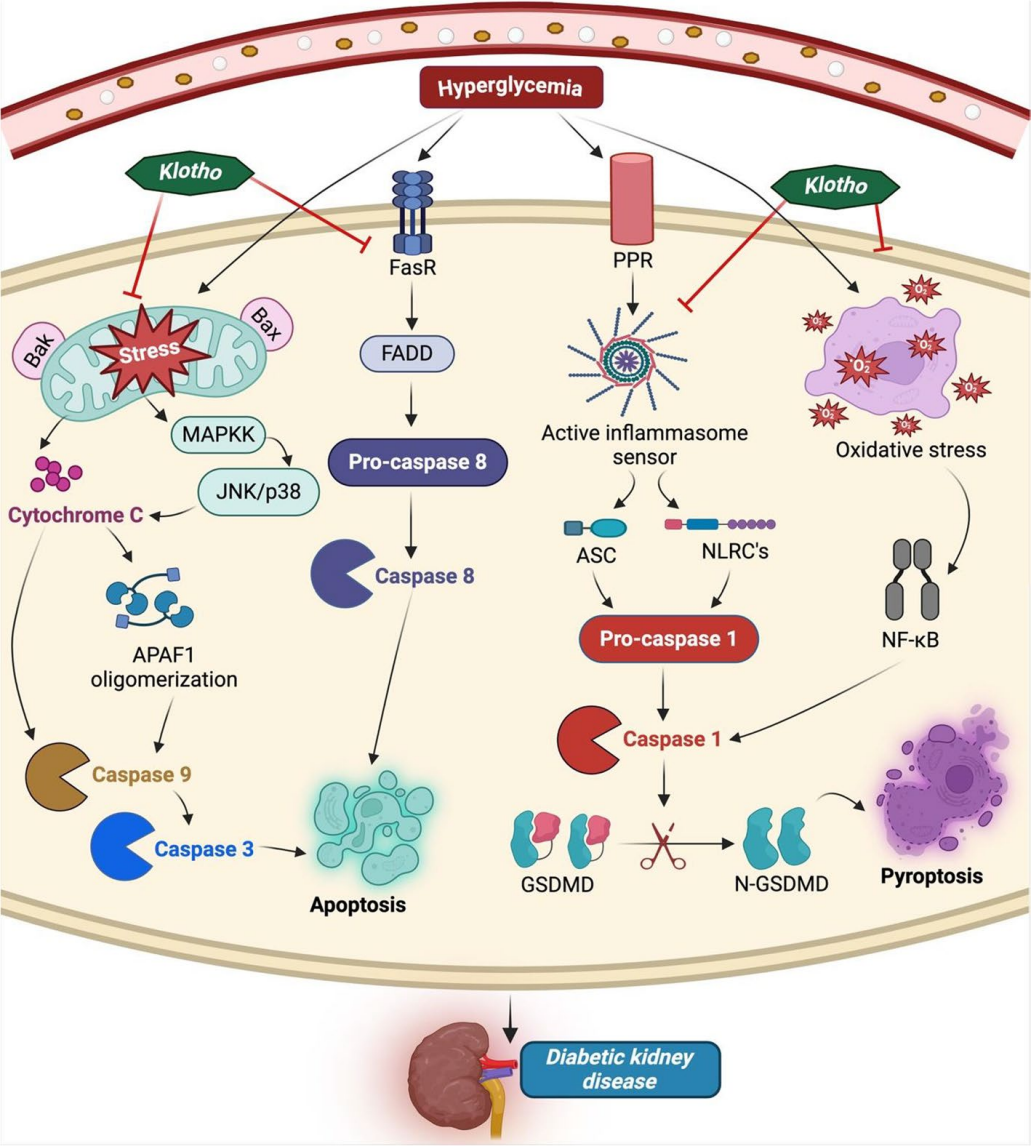
Klotho inhibits apoptosis and pyroptosis pathways implicated in diabetic kidney disease. Mitochondrial stress causes Cyto C synthesis, FasR stimulates FADD, and oxidative stress activates NF-κB, leading to Caspase 3 activation and cell death by apoptosis. Through PPR, activated inflammasome triggers Caspase 1 activity, which cleaves GSMDM, leading to the N and C terminals’ separation and subsequent pyroptosis. However, Klotho shows anti-apoptosis and anti-pyroptotic action in DKD Abbreviations: RAGE, receptor for advanced glycation end-products; MAPKK, mitogen-activated protein kinase kinase; GSDMD, Gasdermin D

Physiologically, the kidney is the key organ to maintain soluble Klotho equilibrium. In DKD, most kidney tubular epithelial cells are damaged, and the remaining cells cannot make and release sufficient soluble Klotho into the circulation. Meanwhile, hyperglycemia plays a role in the development of DKD, where the decline in kidney function is noticeable in disease conditions [61]. Furthermore, the hyperglycemic condition during DKD can cause ROS generation, alter homeostasis parameters, and cellular and glomerular dysfunction, resulting in kidney injury, which triggers various pathological mechanisms, such as oxidative stress, inflammation, fibrosis, apoptosis, autophagy, and mineral metabolism. After much research on Klotho, no complete literature describes its multiple action methods in DKD. As a result, based on available research, we outline the different roles that Klotho plays in the development and progress of DKD in Fig. 1, 2 and Fig. 3.

### Anti-oxidative action

An imbalance of ROS generation and antioxidant levels triggers the oxidative stress phenomenon. It is a risk factor in the kidney and acts as a leading cause of various kidney diseases. Previous research has shown that diabetes people generate more ROS, which leads significantly to the development and progression of DKD [62]. Oxidative stress causes aberrant oxidized low-density lipoprotein (ox-LDL) accumulation, leading to DKD and decreased Klotho expression [25]. Thus, it is essential to understand how Klotho contributes to oxidative stress in kidney-related problems, particularly DKD (Fig. 1).

DKD initiation and advancement appear to be a disruption of Klotho-related cellular activities. Researchers revealed that the reduced amounts of Klotho in DKD cause a shift in cellular lifespan [25, 63]. Crucially, this process is closely correlated with oxidative stress, which drives inflammation. Klotho is an anti-oxidant that saves the kidneys from oxidative stress and assists in expressing antioxidant enzymes [64, 65]. In both in vitro and in vivo models, Klotho functions as a renoprotective, reducing the amount of oxidative stress indicators like ROS and MDA in early-stage DKD [66].

Patients with DKD have higher levels of oxidative stress, which causes downregulation of s-Klotho synthesis and overexpression of nitric oxide, catalase, and superoxide dismutase (SOD) levels [66]. Researchers performed two experiments on kidney disease in mice models. In the first study, Klotho acts as a renoprotective factor by balancing mitochondrial structure, function, wellness, and protection against oxidative overloads [67]. In another, Klotho diminished the activity of lactate dehydrogenase (LDH), and a Klotho deficit was linked to elevated oxidative stress markers [68, 69].

The emergence of oxidative stress in cells includes activating molecular mechanisms that eliminate ROS. Forkhead box O1 (FoxO1) protein manages redox homeostasis, anti-oxidant protein synthesis, oxidative stress, and apoptosis and plays a crucial role in the pathophysiology of DKD. In the kidney cortex of DKD models, FoxO1 expression was reduced [70]. Furthermore, the overexpression of Klotho in DKD resulted in the activation of FoxO1, inhibition of intracellular ROS and mitochondrial superoxide levels, along with the generation of antioxidation activity against high glucose (HG)–induced oxidative stress and apoptosis [71]. In DKD using type 1-like diabetic rats, Klotho protects against DNA damage, reduces ROS and hydrogen peroxide (H_2_O_2_) levels, and promotes kidney Klotho recovery [72]. Oxidative stress refers to an imbalance that occurs between the formation of ROS and the antioxidant defence system; this imbalance is controlled by Klotho, which also maintains the FOXO3a gene expression, preventing ER stress and filtration barrier damage in DKD podocyte injury [72]. All of the research pointed to a possible connection between oxidative stress and Klotho in DKD, indicating Klotho has antioxidant action.

### Anti-inflammatory effects

DKD is one of the leading causes of kidney disease, with kidney inflammation residing as a frequent consequence. Multiple mechanisms, including activation of macrophages, dysfunction of immune cells, accumulation of inflammasomes, and the release of cytokines and chemokines in the kidney, can trigger these inflammatory responses. Hence, controlling inflammation is essential for treating and preventing DKD (Fig. 1).

The Nuclear factor erythroid 2-related factor 2 (Nrf2) signaling pathway plays a crucial role in inflammation by monitoring inflammatory factors and the antioxidant response element (ARE) signaling pathway [73, 74]. However, the increased attention on Klotho has revealed that it boosts the Nrf2 signaling pathway to treat urinary tract-related diseases, including DKD and CKD [44, 75]. Klotho activated Nrf2 signaling in HK2 cell line in sepsis models, which enhanced kidney cell response and significantly lowered the levels of oxidative stress, lipid peroxidation, and inflammatory markers including TNF-α and Interleukins (IL-6) [76].

Elevations in Klotho's levels contribute to the regulation of inflammation by inhibiting the pro-inflammatory response produced by hyperglycemia through the mechanism of NF-κB, which reduces kidney injury [19, 20]. According to investigations, via an NF-kB pathway, indolyl sulphate and TNF-alpha accumulation can increase the production of ROS and decrease the expression of Klotho [77, 78]. Furthermore, epigenetic research has discovered that N6‐methyladenosine (m6A) modification in the Klotho gene suppresses Klotho expression and increases inflammation and kidney damage in diabetic kidney disease [79].

Tubular Toll-like receptor (TLR) 2 and 4 are the primary mediators of DKD, with TLR4 overexpression identified in the kidney tubules of DKD biopsies, which was positively linked with the transcription of pro-inflammatory genes, the formation of inflammatory proteins, and the buildup of ROS [78, 80, 81]. It has been shown that Klotho blocks TLR4, which reduces pro-inflammatory indicators in mesangial cells grown in HG media [82] and also downregulates the inflammation produced by kidney injury via a protein hydrolysis mechanism connected to deglycosylation [78, 83]. Another study found that macrophage inflammatory protein-3 alpha (MIP-3) is a pro-inflammatory chemokine whose signaling pathways enhance inflammation in early DKD [84].

DKD occurs as a result of a prolonged HG diet and glomerular dysfunction. These alterations are accompanied by activating pro-inflammatory markers in the kidneys, with IL playing a significant role. The multifunctional cytokines IL-6 and IL-8, which have pro-inflammatory and pro-fibrotic properties, and their receptors are essential for the onset and progression of kidney disease [85]. Through inhibiting retinoic acid inducible gene I (RIG-I) and NF-κB signaling pathways, Klotho suppresses IL-6 and IL-8, leading to the recovery of podocyte damage and kidney interstitial inflammation [85, 86]. Furthermore, Klotho alleviates cyclosporine A-induced nephropathy in vivo and in vitro models by blocking the PDLIM2/NF-κB pathway, which reduces the level of IL-8, IL-6, and IL-12 [86]. Thus, Klotho may have therapeutic effects by activating anti-inflammatory mechanisms and suppressing pro-inflammatory signaling in DKD.

### Anti-fibrosis

The various signaling pathways involved in the fibrosis mechanism of DKD such as TGF-β1, Wnt, insulin/IGF-1, PI3K-Akt, and Janus kinase/signal transduction and transcription activation (JAK-STAT) whereas the secreted form of Klotho have an inhibitory role against it. These pathways are crucial in the development of myofibroblasts, which in turn leads to fibrosis [87–89]. Glomerular endothelial cells (GEC) are specialized cells in glomerular capillaries that can maintain a high level of filtration process, remove the filtration barrier, and perform a crucial role in DKD. Klotho effectively cures human fibrotic glomerular endothelial cells injured by excessive glucose and reduces DKD in db/db mice model characterized by oxidative imbalance, inflammation, and structural modifications. Furthermore, in comparison with untreated mice, kidney tissue samples collected from DKD animals exhibited significantly decreased Klotho protein expression whereas, RAAS and Wnt1/β catenin signaling-related proteins are elevated which is considerably reversed by rAAV-Klotho injection [90]. Hence, Klotho works as an inhibitor of the RAAS and Wnt/beta-catenin signaling pathway, and a lack of it can lead to DKD.

Klotho suppresses the NF-kB signaling system, which prevents kidney fibrosis caused by increased cytokine expression, macrophage infiltration, inducible NO synthase (iNOS) and cyclooxygenase-2 (COX-2) [91, 92]. Additionally, research findings indicate that the Klotho operates as an antifibrotic in kidney diseases which are mainly linked with the decreased amounts of early growth response 1 (Egr-1) expression through the blocking of TGF-β1/ suppressor of mother against decapentaplegic (SMAD) signaling in HG exposed human mesangial cells [93]. Klotho lowers the expression of TGF-β1 induced by epithelial-mesenchymal transition (EMT) markers in NRK52E cells [89] and also prevents kidney fibrosis in rats by blocking tubular fibroblasts, fibroblast-specific protein-1, and extracellular signal-regulated kinase 1 (ERK-1) signaling pathways [94, 95] (Fig. 1).

IL-6 is a significant factor in kidney illness. It causes the death of interstitial kidney tissue by causing collagen fibres to form, which in turn speeds up the development of fibrotic lesions [96, 97]. In the UUO-induced fibrotic kidneys of mice, Klotho reduced the enhanced mRNA expression of profibrotic markers such as TNF-α, IL-1β, and IL-6 [3]. All of these findings imply that Klotho may potentially be beneficial for fibrosis associated with the formation and progression of DKD.

### Anti-apoptosis

DKD is associated with hyperglycemia and insulin resistance in patients with diabetes. Hyperglycemia may trigger apoptosis in kidney tubular epithelial cells, suggesting glucotoxicity. Research demonstrated that hyperglycemia in mice with type 1 and 2 diabetes causes an increase in nicotinamide adenine dinucleotide phosphate oxidase (NADPH) oxidase activity, elevate ROS production and promotes the enlargement of mesangial matrix and podocyte apoptosis, all of which contribute to DKD. In addition, hyperglycemia prompts oxidative stress production, decreases GFR, and stimulates proapoptotic p38 MAPK, leading to podocyte lytic death in cultured podocytes [21, 98]. These results were reversed when podocytes were cultured in an HG medium and incubated with Klotho protein. The podocyte injury in DKD was significantly attenuated, as evidenced by lowered apoptosis-related protein expression, pro-apoptotic marker levels, cell mortality, and glomerular hypertrophy [72, 99].

Caspases are important in DKD because they are involved in both the initiation and the end phases of apoptosis. The pathophysiology consists of death-inducing ligands, for example, Fas and TNFa, which attach to their receptors and activate caspases 3, 6, and 7, and also release the pro-apoptotic proteins, especially cytochrome c, connects to apoptotic protease activating factor-1 (Apaf-1) and pro-caspase 9 and activates caspase 9, all of these caspases generate apoptotic impact [100, 101] (Fig. 3). In addition, the mitochondrial outer membrane permeabilization (MOMP) is regulated by the family of Bcl-2 proteins, which are composed of either anti-apoptotic (Bcl-2, Bcl-XL, Bcl-w) or pro-apoptotic (Bax, Bak, Bad, Bim). The balance between these proteins modulates the cell apoptosis cycle [102, 103]. Klotho inhibits apoptosis in HG-treated podocytes in DKD via downregulating the ratio of Bax/Bcl2 and caspase 3 expression [104]. Furthermore, Klotho suppresses Caspase3 and Bax expression in podocyte damage in STZ-induced mice [70]. A deficiency of Klotho was observed in STZ-induced diabetic mice, which results in overexpression of pro-apoptotic markers like Nrf 2 and leads to injury to kidney glomeruli, kidney tubules, and podocyte dysfunction, contributing to the pathogenesis of DKD [105].

In recent research, Klotho inhibits oxidative stress and activates the TRPC6 signaling pathway, protecting podocytes against apoptosis caused by HG levels/diabetes in vitro and diabetic mouse models [106]. Moreover, Klotho protects podocytes from injury and apoptosis, thereby improving glomerular filtration function, preventing glomerulosclerosis, and lowering the risk of DKD progression in db/db mice [71]. Considering all these studies, Klotho is likely involved in regulating apoptosis in DKD.

### Autophagy

Autophagy is a crucial cellular mechanism in both health and disease since it is necessary to preserve the homeostasis of glomeruli and tubules. Deficit in autophagy has been linked to several inflammatory illnesses, most notably the development of DKD. It was found that autophagy may be stimulated by intracellular stress, which can be driven by ROS generation, endoplasmic reticulum (ER) stress, hypoxia, DNA damage, release of pro-inflammatory markers, and immunological signaling. Therefore, Autophagy is strictly controlled to help cells adapt to and fight against stress [23, 107]. Furthermore, hyperglycemia-induced modifications in intracellular metabolism and cellular events, including accumulation of AGEs, trigger the RAAS, activate PKC, and disrupt the amount of Klotho protein. These implications have been connected to DKD and suppress vitamin D receptor activity [22].

Autophagy is an essential recycling mechanism that breaks down damaged organelles, accumulates abnormal proteins, and preserves biological processes. According to previous research, Klotho enhanced autophagy via AMPK activation and ERK pathway inhibition (Fig. 2). This resulted in a significant increase in autophagosome count and a reduction in kidney tubular damage in both in vitro and in vivo models of DKD [22]. Autophagy is regulated by nutrient-sensing mechanisms, which include the AMPK pathway, the mTOR signaling system, and the IGF-1 signaling route. To ameliorate diabetes and kidney damage, Klotho controls the elevated level of autophagic flux by blocking the AKT/mTOR pathway or the IGF-1-mediated PI3K/Akt/mTOR system [108, 109].

However, substantial research indicates that autophagy plays a regulatory function in DKD. Long-term exposure to HG levels damages kidney mitochondria and inhibits autophagy in cells, which hastens the onset of DKD [110]. Nonetheless, Klotho can increase autophagy and lessen kidney cell damage from developing. Apart from the previously discussed autophagic routes, Klotho also controls Beclin-1, an essential autophagy regulator, and might potentially modify autophagy activities by changing the proportional amounts of the Beclin-1/Bcl-2 protein complex [111, 112]. Interestingly, Klotho suppresses Beclin-1's connections with additional autophagy-related proteins and enhances Beclin-1's affinity towards Bcl-2, preventing autophagic functioning in DKD [113]. In such consequences, Pi encourages Beclin-1's attachment to BCL-2, the protein that disrupts autophagic flow. Notably, Pi misguides the autophagic events, whereas Klotho modulates them [108]. Because of this, Klotho may manage Pi outflow by controlling autophagic flow.

### Calcium and phosphate metabolism

Calcium and phosphate are essential in forming biological materials, signal transmission, and various physiological activities. A well-known endocrine factor, FGF23, directly targets the kidney phosphate transport mechanism. It monitors the production of parathyroid hormone (PTH), calcitriol, and vitamin D. It is also tied to the kidney-parathyroid gland-bone axis and controls phosphate homeostasis. Endocrine FGF15/19, 21, and 23 lack attraction towards heparin cofactors; they need Klotho proteins to function as co-receptors. When Klotho is present, the binding mechanism is activated, which binds the FGF23 protein to the Klotho/FGFR1c receptor in the kidney tubules [114]. This causes the kidney's load to drop, urine output to rise, and calcium and phosphate balance to be maintained.

On the other hand, hyperphosphatemia is caused by abnormalities in Klotho or FGF23; this seems to indicate that people with type 2 diabetes have a higher risk of developing CKD [115, 116]. Increased Klotho levels and vitamin D supplementation are linked to decreased mortality by reducing the risk of CKD. However, in individuals with CKD, controlling the metabolism of calcium and phosphorus in the presence of vitamin D additionally aids to some extent by raising local Klotho levels and preserving endothelial integrity and functional stability [117, 118].

In DKD patients and mice with STZ-induced DKD, kidney α-Klotho expression was considerably lower, and urine calcium excretion was highly elevated. Additionally, there was a reduction in α-Klotho level in distal convoluted tubules, which might impact urinary calcium excretion in cases of early DKD [119]. Hyperphosphatemia and hypercalcemia are among the conditions associated with age-like syndrome found in Klotho-deficient mice, which indicate an imbalance in phosphate and calcium metabolism. It causes vascular calcification, typified by medial calcification in the aorta and is a condition frequently seen in individuals with DKD [120, 121]. Besides kidney cells, Klotho regulates PTH secretion and calcium homeostasis via the Na + , K + -ATPase transporter in the choroid plexus [122].

Furthermore, to safeguard the calcium homeostasis in the kidney cell line, Klotho stimulates the transient receptor potential vanilloid (TRPV) 5 channel from the internal and external of the cell [123]. TRPC5 and TRPC6 expression is found in podocytes and is important for Ca2 + homeostasis regulation (Fig. 2). Klotho reduces puromycin aminonucleoside (PAN)-induced podocyte death by targeting TRPC6 via miR-30a, which activates calcium/calcineurin signaling and suppresses phosphoprotein phosphatases catalytic subunit Alpha (Ppp3c-α), phosphoprotein phosphatases catalytic subunit beta (Ppp3c-β and Ppp3r1), and nuclear factor of activated T cells-3 (Nfact3) expression [124]. This study demonstrated that TRPC6 is a Klotho target in podocytes in DKD. Although the specific pathway is unknown in DKD, it is evident that Klotho helps regulate the body’s calcium and phosphate balance.

### Pyroptosis

Pyroptosis, also known as caspase 1-dependent cell death, is a particularly inflammatory kind of lytic programmed cell death. According to some recent research, hyperglycemia is linked to certain pattern recognition receptors (PRRs) in cells, which lead to cell enlargement, rupture, and release of contents, proinflammation, and pyroptosis. These effects ultimately result in kidney injury and a loss of kidney function [125]. These pyroptosis signal transduction pathways are closely linked to the pathophysiology of DKD (Fig. 3). A major player in the initiation of pyroptosis is NLRP3, which is a vital indicator of tissue injury. The disruption of inflammasome activation via RP3 is reportedly attributed to the pathophysiology of several metabolic and inflammatory disorders, including traumatic brain injury, alzheimer’s disease, psoriasis, diabetes-related wound healing defects, and kidney injury [126–131].

Additionally, hyperglycemia, hyperlipidemia, and hyperuricemia may trigger the NLRP3 inflammasome-induced pyroptosis. The well-known anti-inflammatory protein Klotho may have a renoprotective impact on kidney disorders by blocking the pyroptosis mechanism. An investigation has shown that Klotho significantly decreased NLRP3 inflammasome by downregulating the activity of caspase-1, IL-1β, Gasdermin D (GSDMD-NT), ROS, and upregulating autophagic cycle in contrast-induced kidney tubular cell pyroptosis in both in vivo and in vitro (Fig. 3) [132]. Pyroptosis is carried out via caspase-dependent GSDMD cleavage. In this mechanism, the cleavage fragment of GSDMD at the NH_2_ terminal enters the plasma membrane. It generates membrane holes, increasing the release of inflammation-associated cytokines (e.g., IL-1b and IL-18), culminating in kidney injury in diabetes [133]. Parallel to this, the experimental findings demonstrated that elevated levels of IL-1β and IL-18 in HK-2 cells were stimulated with HG concentrations. Additionally, pyroptosis-related proteins were found and compared in the kidney tissues of DKD patients and healthy individuals [134].

A critical factor in the development of kidney damage is reduced Klotho, which may be linked to podocyte pyroptosis in DKD. A recent study reported that Klotho regulated podocyte pyroptosis in DKD by suppressing oxidative stress levels, NF-κB activation, NLRP3 inflammasome complex, and improved skeletal injuries, morphological abnormalities, and cell collapse in cultured podocytes exposed to HG [135]. Klotho protects CM-induced HK-2 cells by reducing oxidative stress, preventing NF-κB activation, and downregulating proteins linked to pyroptosis, such as NLRP3, caspase-1, GSDMD, and cleaved-GSDMD [134].

To be precise, pyroptosis, apoptosis, and autophagy are components of programmed cell death. The cysteine-3 GSDME (caspase-3) signaling pathway can transform apoptosis into pyroptosis. Pyroptosis can then be inhibited by autophagy by lowering mitochondrial ROS and blocking NLRP3 inflammatory vesicle activation. Therefore, by disrupting all three signaling pathways, Klotho, which is intimately linked to cell death, can impact the progression of DKD. However, more research is needed to determine the specific mechanism via Klotho that controls cell death in the setting of DKD.

### Uraemic toxin-Indoxyl sulfate

Indoxyl sulphate (IS) is a little protein-binding uraemic toxin (PBUTs), a tryptophan metabolite produced by the colon. During bacterial lysis of indoles, the blood takes tryptophan from the colon. After that, it undergoes oxidation and acidification in the liver to become IS, reaching the bloodstream as an anion ion and binding to albumin [136]. Anion transporters-1 (OAT1) and OAT3 are responsible for excretion from kidney tubules [137]. Thus, kidney function is compromised in patients with kidney disease, OATs are downregulated, serum IS levels are steadily rising, and klotho protein levels fall.

In current times, IS has received a lot of interest as one of the uremic toxins and has a significant role in kidney illnesses such as DKD, CKD, kidney tubule cell destruction, kidney fibrosis [138], and other conditions like coronary fibrosis, vascular calcification, and atherosclerosis [139, 140]. According to studies, IS can induce cell senescence and downregulate the expression of Klotho in proximal kidney tubule cells by triggering NF-κB through the generation of ROS in the kidney of hypertensive rats [141]. In patients on continuous dialysis, the serum IS concentration steadily raised to its maximum level, whereas the Klotho protein content steadily declined [142]. Patients with CKD have been shown to excrete less IS due to having a lower GFR [143]. The buildup of IS can impact kidney and heart function, leading to kidney fibrosis in CKD patients as well as encouraging inflammation and cardiac hypertrophy [141, 144].

Alternatively, it has been demonstrated that by promoting M1 polarisation, downregulating klotho may hasten the development of DKD [145]. IS increases the production of pro-inflammatory cytokines (TNFα, IL-6, and IL-1β), decreases Klotho expression in THP-1-derived macrophages, and enhances M1 macrophage polarization [141]. In vitro and in vivo, Klotho overexpression reduces kidney damage and heart failure by inhibiting the IS-induced inflammatory response macrophage polarisation by raising the anti-inflammatory factor IL 10, lowering pro-inflammatory cytokines, and deactivating the NF-κB pathway [141]. Therefore, increased serum IS levels and decreased Klotho protein levels are independent risk factors that may be used to treat kidney disease.

## Role of Klotho in other diseases

Klotho, first identified as an anti-aging protein, has attracted considerable attention due to its multifaceted roles in human health and illness. Beyond its well-known implications in phosphate metabolism and oxidative stress reduction, Klotho has emerged as a critical regulator in various pathological conditions. Klothos’ systemic effects underscore its therapeutic diversity, ranging from promoting neural resilience in alzheimer’s disease to preventing vascular calcification in cardiovascular diseases [146, 147]. Its ability to regulate inflammation, oxidative stress, and cellular signalling positions it as a promising candidate for addressing chronic and ageing-related disorders. It has been reported to reduce inflammation in inflammatory bowel disease, protect the liver from fibrotic and metabolic damage, and exhibit tumour-suppressive effects in cancer [148–150].

Therefore, this section emphasizes expanding the role/functions of Klotho in diseases including cardiovascular disease (CVD), alzheimer's disease (AD), cancer, inflammatory bowel disease, and liver disease beyond DKD, highlighting its potential for revolutionizing treatment strategies for treating intricate, multifactorial illnesses.

### Cardiovascular diseases (CVD)

**Fig. 4:**
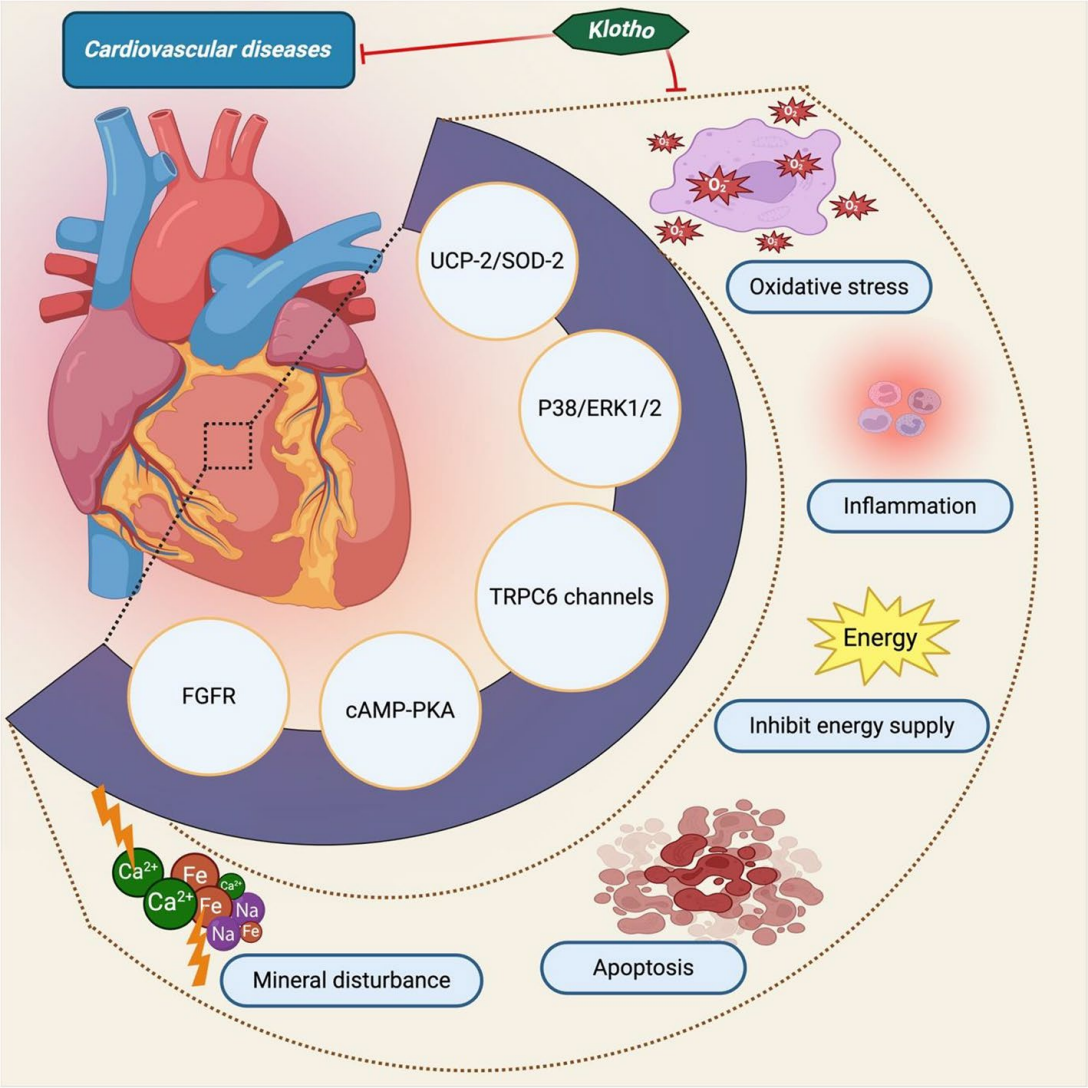
Klotho’s impact on cardiovascular disease (CVD). Cardiovascular disease (CVD) is a societal pandemic. Klotho deficiency, which causes kidney damage, is constantly linked to harmful consequences such as oxidative stress, apoptosis, inflammation, blocked energy supply, and disturbed mineral balance in cardiovascular disease. On the other hand, Klotho overexpression can reduce mortality and morbidity by blocking all of these effects through the TRPC6 channel, P38/ERK1/2, UCP-3/SOD-2, cAMP-PKA, and FGFR Abbreviations: UCP-2, Uncoupling protein 2; FGFR, fibroblast growth factor receptor; PKA, protein kinase A; SOD-2, Superoxide dismutase 2

In recent years, significant attention has been shown to Klotho protein and various disorders. Researchers are working to determine the functions of Klotho and how it contributes to the dysfunction of the most common illnesses, including cardiovascular diseases (CVD) (Fig. 4). The precise mechanisms by which klotho benefits cardiovascular and kidney functioning remain ambiguous. However, several possibilities have been proposed. Firstly, several in vitro and in vivo investigations have demonstrated that α-klotho possesses anti-inflammatory, anti-fibrotic, and anti-thrombotic effects on vascular endothelium that may control impaired endothelial function [151]. Secondly, by reducing cardiovascular calcification linked to CKD mineral and bone abnormalities, Klotho plays a significant role in maintaining calcium and phosphorus balance [152]. However, several cohort studies have demonstrated an opposite relationship between Klotho expression and coronary calcium level in patients with CKD [153]. Thirdly, In animal models of ischaemic kidney damage, supplementation of exogenous klotho has been shown to ameliorate glomerular and tubular harm, indicating that α-klotho performs various renoprotective functions [154]. Fourthly, the lack of Klotho seems responsible for kidney fibrosis and cellular mortality, primarily caused by the epithelial to mesenchymal undergoing change [155]. However, the assessment of this link may be made more challenging by unforeseen factors like elevated blood pressure, coronary artery disease, smoking, and lack of exercise that contribute to both Klotho insufficiency and severe kidney or cardiovascular performance [156, 157].

In the first study on plasma Klotho in CVD, typical cardiovascular risk variables were observed, including age, gender, cholesterol, blood pressure, and diabetes. Remarkably, a drop in plasma Klotho levels was associated with an increased risk of CVD in adults [158]. Likewise, a correlation was seen between plasma Klotho and atherosclerosis. The findings indicated that a reduced serum Klotho level was linked to a broader epicardial fat and carotid artery thickness and delayed flow-mediated dilatation of the brachial artery [159]. Disturbances in serum phosphate and calcium levels lead to hyperphosphatemia, the main factor in the onset and advancement of CVD (Fig. 4). Compared to CKD mice, Klotho deficiency causes worsening CVD and an elevated blood phosphate level. However, Klotho supplementation or overexpression can lower serum phosphate levels, enhance the kidney's function, and prevent CVD [91, 159, 160]. Apoptosis and oxidative stress play a key role in the development of CVD. The study discovered that Klotho gene transfer dramatically reduced superoxide generation by blocking the cAMP-PKA pathway and lowering the protein level of Nox2 expression in vascular smooth muscle cells (VSMCs) [160]. Exogenous Klotho supplement has been shown to reduce ROS production brought on by salusin-β, lower the progression of CVD, and enhance heart functions [161]. Additionally, Klotho can prevent AngII-induced VSMC death and suppress mTOR signaling induced by rapamycin, improving CVD and guard against vascular disease in CKD [162].

Cardiovascular disease is the primary cause of mortality in kidney-related illnesses patients, and meta-analysis study offers an in-depth and extensive examination of the relationship between Klotho levels and negative cardiovascular results. In 200 hemodialysis patients, lower levels of α-klotho were linked to an increased risk of peripheral vascular disease, a higher incidence of cardiovascular events (such as myocardial infarction or stroke, either fatal or non-fatal), and a higher risk of death from all causes [163]. The National Health and Nutrition Examination Survey, which involved 9870 participants from the US, found that a lower klotho is linked to a significantly higher risk of CVD mortality. Nevertheless, the biggest risk of all-cause and CVD mortality was related to vitamin D metabolism alteration, which was reached by the combination of low blood 25-hydroxy vitamin D and low klotho [147]. Patients with maintenance hemodialysis (MHD), Klotho, and CVD mortality are strongly connected. Low KL values help predict CVD mortality in MHD patients with minimal vascular calcification [164]. A meta-analysis of fourteen studies found that individuals with low Klotho levels had a greater risk of cardiovascular mortality and CKD development, with low, moderate, or high heterogeneity [165]. To sum up, decreased klotho levels are a strong indicator of unfavourable consequences, such as elevated risks of cardiovascular and all-cause mortality as well as the advancement of kidney diseases.

### Alzheimer’s disease (AD)

Alzheimer's disease (AD) is a neurodegenerative condition characterized by neuroinflammation, mitochondrial dysfunction, extracellular amyloid-beta (Aβ) plaque, and neurofibrillary tangle accumulation inside cells. These factors gradually impair cognitive function and destruction of nerve cells with increasing age [166–169]. The development of senile plaques composed of β-amyloid (Aβ) peptides is a characteristic component of AD aetiology. Amyloidogenic activation of the amyloid precursor protein (APP) produces β-amyloid (Aβ) peptides, as well as building of the interior region of APP and released ectodomain segments (APPsα and APPsβ) [170]. While the exact physiological role of APP remains unclear, its overexpression demonstrates a neurotrophic impact on the viability and proliferation of brain cells.

Klotho, a measure of lifespan, decreases with age, diabetes, kidney failure, neurological diseases (AD, Parkinson's disease (PD), stroke, amyotrophic lateral sclerosis, epilepsy, glioblastoma multiforme). It is abundantly expressed in the kidneys and detected in the brain, notably in the neurons, Purkinje EC cells, choroid plexus, cerebrospinal fluid (CSF), and cerebral white matter [171]. According to experimental research, systemic Klotho increase could improve synaptic plasticity, cognitive function, and neural resistance to aging, PD, and AD [146]. In the cortex and hippocampus, Klotho can modulate synaptic GluN2B levels, which leads to an elevation of NMDAR-dependent genes, particularly Fos, that are involved in building up memories [172]. By activating NMDAR, klotho enhances long-term potentiation (LTP), an essential component of learning and recall [173]. In vitro models of neurodegeneration frequently employ Aβ and L-glutamate as oxidative stimulators, and Aβ's neurotoxic action is also linked to cellular damage brought on by ROS generation. Klotho protects from L-glutamate and oligomeric Aβ-induced neurotoxicity in hippocampal regions most susceptible to oxidative stress [174]. Furthermore, a study suggested that exterior APP processing generates APP ectodomain segments that enhance Klotho expression and confer shielding against Aβ neurotoxicity throughout aging. Thus, Klotho serves as a physiological target of APP, regulating neuronal health and development [175].

**Fig. 5:**
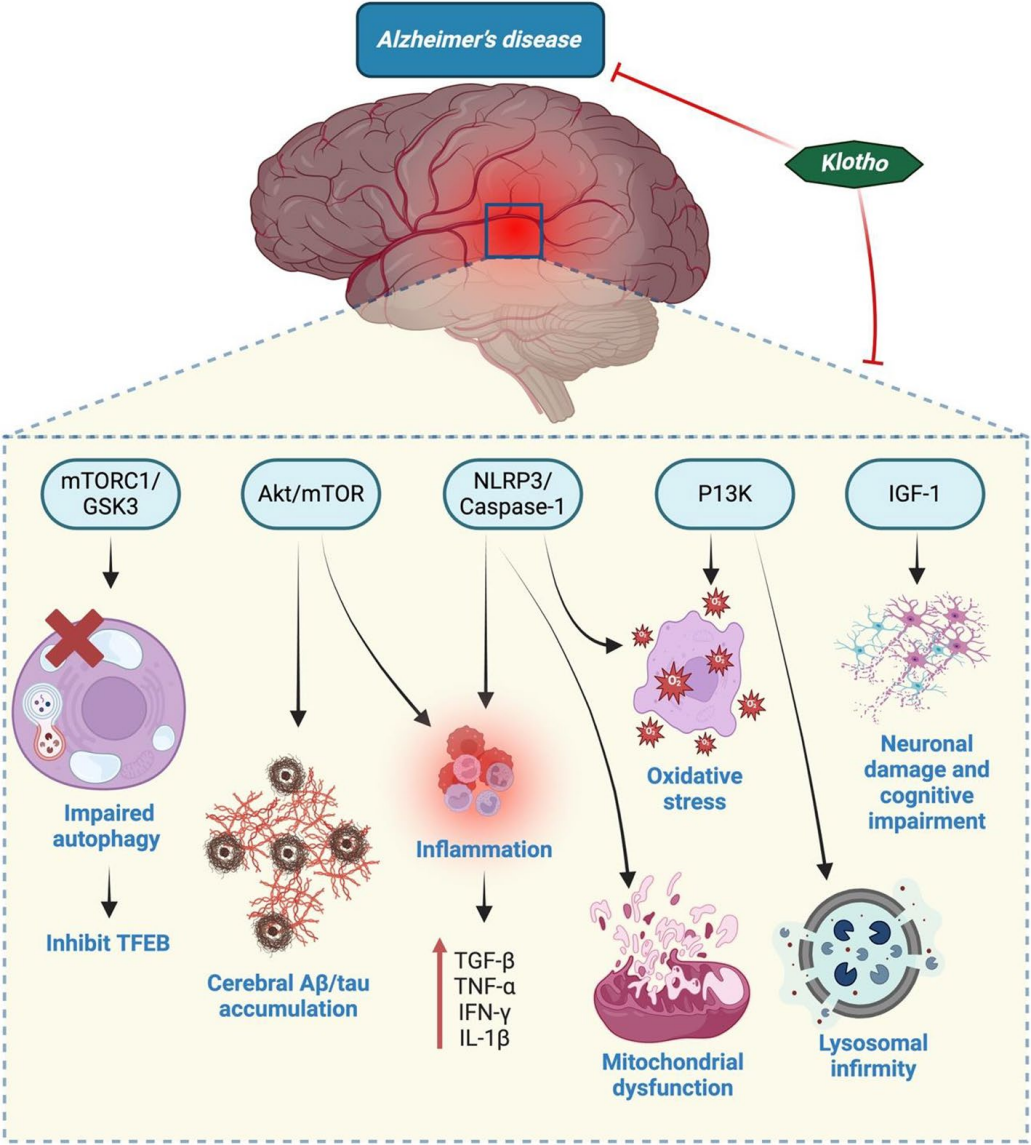
Effect of Klotho on the pathophysiology of AD. IGF-1 stimulation causes cognitive impairment and neuronal damage in AD, while autophagy disruption and TFEB inhibition are caused by mTORC1/GSK3 activation. However, klotho prevents AD by blocking PI3K, NLRP3/Caspase-1, and Akt/mTOR, which in turn prevents inflammation, oxidative stress, cerebral Aβ/tau buildup, and mitochondrial and lysosomal dysfunction. Moreover, it suppresses mTORC1/GSK3 and IGF-1 Abbreviations: TFEB, transcription factor EB; GSK3, Glycogen synthase kinase 3

Klotho, a single-pass transmembrane protein predominantly in the kidney and brain, is implicated in several metabolic pathways regulating aging and neurodegeneration. Research has shown that lower levels of Klotho expression are found in the cerebral regions of patients with early-stage AD and aging individuals [176, 177]. In neural cells, the Tau protein stabilises and preserves the strength and framework of microtubules. On the other hand, AD causes harm to neurons and cognitive impairment due to the hyperphosphorylation and intracellular accumulation of the Tau protein in the neurons [168] (Fig. 5). A recent study on AD patients amply illustrated Klotho's neuroprotective impact by reducing Tau-related symptoms. PET imaging revealed improved cognitive function and lowered intense Tau symptoms [178]. Elevated Klotho expression helps ligustilide's neuroprotection in a mouse model of AD by inhibiting the insulin/IGF-1/mTOR signaling pathway, resulting in activation of the transcription factor FoxO to reduce oxidative stress and also enhance brain cognitive function [175, 179]. In the APP/PS1 mice model of alzheimer's disease, Klotho upregulation boosts amyloid clearance and cognition by inhibiting NLRP3, encouraging microglia transformation, and managing the expression of the Aβ transporter, ultimately improving Aβ clearance in the brain [180]. Neuronal cells require autophagy to eliminate intracellular toxins and damaged organelles that build with age. Klotho protein can promote autophagy induction, which enables the removal of neurofibrillary tangles (NTFs) and regulates Aβ homeostasis [181]. Furthermore, Klotho protein drives up TFEB-mediated lysosomal gene transcription and promotes Transcription factor EB (TFEB) translocation by blocking mTORC1 and GSK3 kinase activity, hence facilitating autophagy clearance and lysosomal activity [182]. New evidence has raised the possibility that Klotho may be an anti-AD target protein.

Individuals with AD had markedly reduced cerebrospinal fluid (CSF) Klotho concentrations compared to those with normal cognitive function. Additionally, it revealed that elderly people had lower CSF Klotho concentrations than younger people. A surprising finding showed that women's CSF has lower Klotho levels than men's. Although the observed variation may be due to biological or societal factors, as well as the increased risk and occurrence of the illness related to women, this observation could help women have a greater possibility to develop AD than males [12]. Based on accumulating evidence, the neuroprotective protein Klotho is currently exhibiting encouraging treatment for AD.

### Cancer

**Fig. 6:**
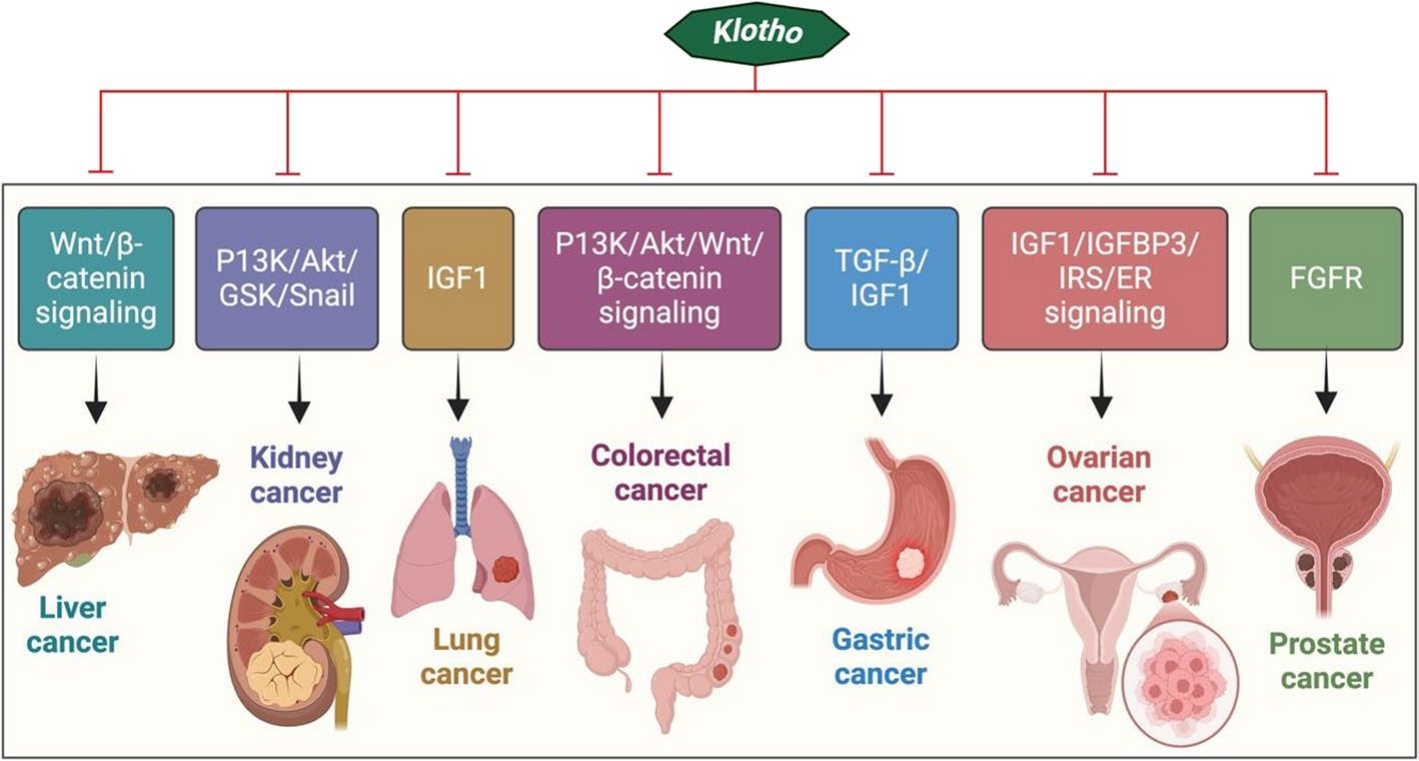
Role of Klotho on cancer biology. Klotho was first identified as an anti-aging protein, but its possible therapeutic use in cancer biology has subsequently attracted a lot of attention. Klotho is involved in a number of malignancies, including those of the lung, liver, kidney, colon, stomach, ovary, and prostate. By altering downstream oncogenic signaling pathways, such as insulin/IGF1, FGF, PIK3K/AKT, TGFβ, p53/p21, and Wnt signaling, Klotho suppresses tumours

In recent times, Klotho's role in carcinogenesis, cancer development, and prognosis has garnered heightened interest. α-Klotho is linked to a tumour suppressor [183, 184], β Klotho is related to both tumorigenic and tumour-suppressive actions in several cancers [185], exhibiting more complicated activities, and γ Klotho expression has been investigated in different forms of cancer [186]. α Klotho suppressed the phosphatidylinositol-3-kinase (PI3K) pathway, which governs cellular development, survival, and multiplication and is activated by many membrane-bound receptors [187] (Fig. 6). β Klotho indicated that FGFR signaling mediates tumor aggressiveness in hepatocellular carcinoma [188]. On the other hand, it suggested acting as a tumour suppressor in pancreatic and breast malignancies [188, 189]. The National Health and Nutrition Survey (NHANES) data from a large population research was used for the first time to analyze the relationship between serum Klotho and cancer. The results indicated that serum Klotho had a negative correlation with cancer but not with tumor-specific death [190]. Klotho was found to be a carcinogen inhibitor in pancreatic ductal adenocarcinoma in three in vivo studies, including a new genetic mouse model and bioinformatics analysis. Additionally, a distinct Klotho DNA hypermethylation pattern appeared in pancreatic tumours [191]. Gene expression studies reveal that lung cancer tissue specimens and cell lines have lower levels of klotho expression in contrast to healthy lungs [150, 192]. According to a recent study, genetic changes in egyptian colorectal cancer (CRC) patients resulted in lower expression of Klotho gene variants (rs1207568 and rs564481) [193]. Given that prior studies have linked low levels of the klotho protein to an increased risk of cancer. Hence, Klotho protein may be a target for oncotherapy.

### Inflammatory bowel disease (IBD)

A collection of long-term illnesses known as inflammatory bowel disease (IBD) results in intestinal inflammation. Crohn's disease and ulcerative colitis (UC) are the two primary forms of IBD. UC is a long-term IBD that can impact the body's large intestine and colon. It is associated with a widespread inflammatory process. Klotho expression in mice was found to be normal. Normal mice exhibit Klotho in the stomach, primarily in the myenteric plexus. In contrast, loss of Klotho results in reduced cajal interstitial cells and their precursors, leading to stomach motor impairment in homozygous Klotho^−/−^ mice [194]. Klotho-deficient mice develop early aging syndrome, whereas normally aging rats have Klotho expression in the gastrointestinal mucosa in epithelial and vascular compartments which is identical to juvenile rats [195]. The study reported suppression of Klotho expression in three mice models, including the TNBS colitis model, the IL-10 deficient colitis model, and the immunological model of IBD [196]. In this study, a typical pattern of intestinal inflammation linked to mucosal injury, disrupted mineral homeostasis, downregulation of vascular defence, and infiltrations of lymphocytes and neutrophils. Moreover, In mpkDCT4 cells, pro-inflammatory markers (IFN-γ and TNF) increased, but Klotho expression reduced [196].

Crohn's disease may harm any portion of the GI tract, including the mouth and anus. Numerous genetic variables linked to the susceptibility of Crohn's disease are inflammatory markers, such as IL-23, which may impact α-Klotho expression levels affected explicitly by one or more genetic factors relating to Crohn's disease. On the other hand, the correlations between circulating Klotho, LDL-C, and Crohn's disease susceptibility may not be affected by endocrine FGFs [148]. Klotho may have a broader function in controlling inflammation; it has been demonstrated that the increase in Klotho expression significantly decreases a number of inflammatory conditions, such as IBD.

### Liver disease

There is little information available on the connection between Klotho and liver disease. In the brain and liver, α-Klotho functions as an antioxidant effector by modifying the apoptotic signal-regulating kinase 1/p38MAPK pathway, which leads to decreased ROS production [197, 198]. According to current knowledge, changes in the Klotho gene may be linked to liver damage in kids with non-alcoholic fatty liver disease (NAFLD) [199, 200], and by controlling the breakdown of energy and adversely influencing the Wnt/b-catenin signaling pathway, Klotho can stop the advancement of liver cancer and decrease obesity. Furthermore, compared to controls, one study indicated that children with alcoholic cirrhosis had considerably greater Klotho levels [201]. However, no study has yet to find a link between Klotho and NAFLD in an extensive sample.

In diet-induced obesity (DIO) mice, Klotho administration reduces adipose tissue and hepatic lipid buildup, mainly decreasing obesity by blocking acetyl-CoA carboxylase (ACC), which increases fat oxidation [202]. In mice lacking Klotho expression, gluconeogenic gene activity is increased in the liver, but non-shivering thermogenesis and uncoupling protein 1 gene expression are compromised [203]. Additionally, atrophy was observed in Klotho-deficient mice’s liver and adipose tissue [60, 203]. In contrast, one investigation revealed that alcoholic cirrhosis is associated with elevated levels of α Klotho and FGF-23, which are also linked to abnormal liver function [204]. Therefore, more research is required to evaluate Klotho's behaviour in liver illness to identify the specific mechanism.

## Recent therapeutic potential of exogenous Klotho in diabetic kidney disease and other diseases

Alterations in the morphology and operating system of the glomeruli in the kidney define DKD. During DKD and DM, Klotho helps to protect the kidney cells from harm, but the expression of Klotho is significantly decreased in kidney tubules of both humans and animals with DKD [205]. Thus, the destruction of kidney function and loss of endogenous Klotho expression might be avoided by exogenous Klotho administration.

Kidney tissue has an abundance of mitochondria and requires a considerable quantity of ATP for physiological stability, implying that mitochondrial health matters greatly for maintaining kidney function. Kidney mitochondrial dysfunction may result in aberrant expression of many signaling pathways that significantly impact the pathophysiological development of DKD [109, 206]. Klotho rescued mitochondria from DKD in db/db mice by stimulating the AMPK-PGC1a pathway and inhibiting mTOR/TGF-β and ROS generation, indicating a renoprotective effect through mitochondrial dysfunction [207]. Study finds that reduced endogenous and exogenous Klotho synthesis and activity in diabetes circumstances trigger cascading pathways, including ERK1/2, p-38, and cell cycle arrest via PPAR-γ, which ultimately results in diabetic kidney disease. After supplementing with recombinant Klotho, compensate for the Klotho loss and recovery from DKD observed [104]. The exogenously supplemented Klotho in cultured kidney cells suppressed TNF-α stimulation and NF-κB activation, thereby minimizing kidney damage and mitigating the advancement of diabetic kidney disease. Therefore, Klotho protein is a highly attractive biomarker, and exogenous Klotho supplementation represents a potentially cutting-edge treatment approach for DKD.

Furthermore, Klotho possesses multiple activities and is majorly expressed in the kidney. A decrease in Klotho has been found in the kidneys of patients with DKD. It has been reported that streptozotocin (STZ)-induced diabetes in rats lowers the kidney Klotho levels, which is related to kidney damage [2]. Klotho deficient in Klotho mutant mice exacerbated STZ-induced elevations of urinary albumin, blood urea nitrogen (BUN), mesangial matrix enlargement in kidney glomeruli, decreased GFR, and kidney hypertrophy, suggesting that Klotho gene deficit may render kidneys more vulnerable to diabetic damage [208]. Thus, Klotho keeps the kidney's activity and morphology safe. In NRK-49F cells, exogenous Klotho supplementation inhibits HG-induced fibronectin protein expression, collagen synthesis, TGF-β signaling, and cell hypertrophy [209]. Exogenously administered Klotho inhibited TNF-a's ability to bind to its receptor in HK-2 cells. It prevented NF-κB activation and the release of pro-cytokines, including MCP-A and IL-8, but it could not prevent phosphorylation [210]. In cultured rat podocytes, the addition of exogenous Klotho in HG media markedly changed the levels of proteins such as Smad2/3, p-Smad2/3, Smad7, and NADPH Oxidase (NOX4) and dropped the production of ROS. Similarly, In DKD rats, kidney damage was prevented by administering exogenous Klotho [211]. In addition, supplementing with exogenous Klotho protein suppresses p-Akt, p-mTOR, and ROS and reduces kidney production of TGF-β, TNF, and fibronectin. These effects mitigate kidney damage and the RAAS in db/db mice, which could be more morally beneficial for patients with diabetes [116].

Exogenous Klotho supplementation regulates glucose production by blocking the glucogenesis pathway and reversing insulin resistance in individuals with type 2 diabetes, averting fibrosis pathophysiology, pro-inflammatory indicators, and oxidative stress, and shielding the kidney from injury [55, 212]. A further investigation demonstrated that Exogenous Klotho supplementation restored the downregulated Klotho expression in humans and animals with DKD and enhanced kidney function [116, 205]. Therefore, several findings support the potential safeguarding function of exogenous Klotho in DKD and various kidney diseases. The recombinant Klotho delivery has shown promise in pre-clinical trials, while safety complications are unlikely to be a substantial barrier. Thus, it's necessary to find valid visible signs for Klotho therapy based on animal studies before starting clinical investigations. Hence, recombinant Klotho is a potentially effective technique for managing DKD and other kidney complications.

Exogenous Klotho supplementation delays the development of CKD-accelerated atherosclerosis through reduced vascular calcification and lessening heart failure [160]. Symptoms of kidney failure can be significantly reduced, and the morbidity and mortality of CVD consequences can be decreased by either genetically overexpressing α-Klotho or by taking an exogenous supplement of the protein [213]. Salusin-β-induced oxidative stress production and vascular calcification (VC) formation have also been shown to be reduced by exogenous Klotho supplements [161]. Furthermore, In the rat model, exogenous Klotho restored sirtuin1 (SIRTI) reduction and minimized the level of cardiac hypertrophy indicators, whereas, in H9c2 cells, it reduced ROS generation, prevented apoptosis, and improved mitochondrial dysfunction [214]. It has been demonstrated that exogenous injection of recombinant Klotho reduces lung fibrosis ex vivo by regulating the expression of known fibrotic genes, extracellular matrix formation, and bronchial fibroblast stimulation [215]. According to one research, exogenous Klotho administration decreased the development of AKD damage to CKD or attenuated ischemic kidney damage. It has also been shown to attenuate glomerular and tubular injury in animal ischemia or immune-complex kidney damage models [165]. In human colorectal adenocarcinoma cells (HT-29), exogenous Klotho may be a helpful therapy due to the elimination of apoptosis resistance and lower tumour activity [216]. Because current DKD and other illness treatment regimens are insufficient, exogenous Klotho supplement may be a revolutionary approach to treating all diseases.

## Conclusion and future perspective

The discovery of new therapeutic approaches that slow down the progression of lesions and improve the quality of life of kidney patients continues to be a challenge for nephrologists and the scientific community. So, Klotho may be an early biomarker and a potential therapeutic target in DKD as well as other specific diseases (CVD, AD, cancer, IBD, liver disease, and aging). Current evidence suggests that Klotho can ameliorate DKD through various mechanisms, including antioxidative, anti-inflammatory, anti-fibrosis, anti-apoptosis, autophagy, pyroptosis, and regulation of calcium and phosphate levels. Numerous efforts have been made to identify the mechanisms of DKD and other specific diseases, and indeed, significant progress has been made. Growing studies strongly pose the potential utility of endogenous Klotho restoration or exogenous Klotho replacement as therapeutic options in DKD and other specific diseases. Exogenous Klotho administration is efficacious in animal studies, but prior to launching clinical trials, many further studies are still needed.

The physiological function of Klotho and associated molecular processes have received enough research in recent years. Nevertheless, there are still a number of unanswered issues about its consequences. For example, 12 weeks after creating a diabetic mouse model, a study discovered that the blood glucose levels in the Klotho overexpression group differed considerably from those in the control group [217]. Nevertheless, it is still unknown if klotho can directly affect blood glucose levels. Apart from preventing Klotho downregulation or substituting klotho that is lacking, focussing on microRNAs has been found to be a successful method of adjusting Klotho levels [218]. However, preclinical research is currently being conducted in this field. Additionally, Klotho may be a useful biomarker for predicting diabetes, endocrine disorders, or aging. Nonetheless, its diagnostic standards and measuring methods require improvement.

Recent research indicates that Klotho is a viable and promising therapy option for kidney illness. On the other hand, targeting Klotho with already available drugs may be an effective DKD therapeutic technique. However, more investigation is required to ascertain the health risks of long-term and high-dose administration of recombinant Klotho or Klotho supplements. Hence, The effectiveness of Klotho in treating DKD has been assessed in a number of clinical trials. Lastly, even though animal models provide the basis of a large portion of the basic research on Klotho, further data from tests in healthy humans or DKD patients is needed to improve our comprehension.

## Data Availability

Not applicable.

## Abbreviations

DKD: Diabetic kidney disease
DM: Diabetes mellitus
CKD: Chronic kidney disease
ROS: Reactive oxygen species
RAS: Renin-angiotensin system
ACE: Angiotensin-converting enzyme
NF-κB: Nuclear factor kappa B
MAPK: Mitogen-activated protein kinase
AGE: Advanced glycation end-products
RAAS: Renin-angiotensin-aldosterone system
PKC: Protein kinase C
GFR: Glomerular filtration rate
VEGF-A: Vascular endothelial growth factor – A
IGF-1R: Insulin growth factor -1 receptor
PI3K: Phosphatidylinositol 3-kinase
mTOR: Mammalian target of rapamycin
IGF: Insulin-like growth factor
TGF β1: Transforming growth factor β1
TNFγ: Tumour necrosis factor gamma
Ox-LDL: Oxidized low-density lipoprotein
LDH: Lactate dehydrogenase
FoxO1: Forkhead box O1
HG: High glucose
TLR: Toll-like receptor
GEC: Glomerular endothelial cells
Smad: Suppressor of mother against decapentaplegic
TRP: Transient receptor potential
PRR: Pattern recognition receptors
STZ: Streptozotocin
CVD: Cardiovascular disease
AD: Alzheimer disease
PBUT: Protein-binding uraemic toxins
IS: Indoxyl sulphate
NAFLD: Non-alcoholic fatty liver disease
IBD: Inflammatory bowel disease
UC: Ulcerative colitis
APP: Amyloid precursor protein
CSF: Cerebrospinal fluid
